# COMMUNITY MEMBERS' PERCEPTIONS OF MASS DRUG ADMINISTRATION FOR CONTROL OF LYMPHATIC FILARIASIS IN RURAL AND URBAN TANZANIA

**DOI:** 10.1017/S0021932015000024

**Published:** 2015-03-19

**Authors:** WILLIAM J. KISOKA, BRITT PINKOWSKY TERSBØL, DAN W. MEYROWITSCH, PAUL E. SIMONSEN, DECLARE L. MUSHI

**Affiliations:** * National Institute for Medical Research, Dar es Salaam, Tanzania; † Tumaini University, Kilimanjaro Christian Medical College, Moshi, Tanzania; ‡ Global Health Section, Department of Public Health, University of Copenhagen, Denmark; § Department of Public Health, University of Copenhagen, Denmark; ¶ Department of Veterinary Disease Biology, University of Copenhagen, Denmark

## Abstract

Lymphatic filariasis is one of several neglected tropical diseases with severely disabling and stigmatizing manifestations that are referred to as ‘neglected diseases of poverty’. It is a mosquito-borne disease found endemically and exclusively in low-income contexts where, concomitantly, general public health care is often deeply troubled and fails to meet the basic health needs of impoverished populations. This presents particular challenges for the implementation of mass drug administration (MDA), which currently is the principal means of control and eventual elimination. Several MDA programmes face the dilemma that they are unable to attain and maintain the required drug coverage across target groups. In recognition of this, a qualitative study was conducted in the Morogoro and Lindi regions of Tanzania to gain an understanding of community experiences with, and perceptions of, the MDA campaign implemented in 2011 by the National Lymphatic Filariasis Elimination Programme. The study revealed a wide variation of perceptions and experiences regarding the aim, rationale and justification of MDA. There were positive sentiments about the usefulness of the drugs, but many study participants were sceptical about the manner in which MDA is implemented. People were particularly disappointed with the limited attempts by implementers to share information and mobilize residents. In addition, negative sentiments towards MDA for lymphatic filariasis reflected a general feeling of desertion and marginalization by the health care system and political authorities. However, the results suggest that if the communities are brought on board with genuine respect for their integrity and informed self-determination, there is scope for major improvements in community support for MDA-based control activities.

## Introduction

Lymphatic filariasis, a devastating disease caused by filarial nematode worms and spread by mosquitoes, is found in more than 80 countries (WHO, 2010; Simonsen *et al.*, 2014). The infection damages the human lymphatic system and its acute and chronic complications cause considerable disability in affected individuals. An estimated 40 million people are incapacitated or disfigured with swollen genitalia (hydrocoele) or dramatically thickened limbs, with hard, rough and fissured skin (lymphoedema/elephantiasis) (WHO, 2013). Affected individuals suffer social and economic consequences due to stigma and reduced productive capacity resulting from these complications (WHO, 2013).

The World Health Organization, through its Global Programme to Eliminate Lymphatic Filariasis (GPELF), has targeted the disease for elimination as a public health problem by 2020 through a dual strategy of mass drug administration (MDA) to interrupt transmission of infection and morbidity control to alleviate disability of people affected by the acute and chronic manifestations (Gyapong *et al*., 2005; Ottesen, 2006; Kyelem *et al.*, 2008; WHO, 2010, 2013). The MDA involves administering drugs to entire endemic populations regardless of individuals' infection status through the use of a two-drug combination of either diethylcarbamazine or ivermectin combined with albendazole once every year. A high treatment coverage of 65% in the endemic population sustained for 5–6 years is required to effectively clear infected individuals and stop the transmission (Michael *et al.*, 2004; WHO, 2011). When a large proportion of the population is not included, or refuses participation, in MDA, a potential reservoir for the parasite is left untreated, thus opening the door to recrudescence of transmission (Talbot *et al.,* 2008; Boyd *et al.*, 2010). A major challenge facing many MDA programmes for lymphatic filariasis control has been to attain and sustain the high treatment coverage required to interrupt transmission in endemic communities (e.g. Gunawardena *et al.*, 2007; Amarillo *et al.*, 2008; Babu & Mishra, 2008; Njomo *et al.*, 2012; Offei & Anto, 2014). A complex set of individual, societal and health system factors has been given as an explanation for the failure of the intervention to reach the optimal drug coverage required to interrupt lymphatic filariasis transmission (Krentel *et al.*, 2013). It is important to identify and understand these factors in order to strengthen the programme approach and increase treatment coverage.

Lymphatic filariasis is widespread in Tanzania, and coastal areas are characterized by high levels of infection and disease (e.g. McMahon *et al.*, 1981; Minjas & Kihamia, 1991; Meyrowitsch *et al.*, 1995; Simonsen *et al.*, 1995, 2002; Rwegoshora *et al.*, 2005). It is estimated that over 34 million individuals live in endemic foci in Tanzania and that 5–6 million are affected by one or more clinical manifestations of lymphatic filariasis (Malecela *et al.*, 2009).

In Tanzania, lymphatic filariasis control is co-ordinated under the integrated Neglected Tropical Disease (NTD) Control Programme, which also oversees MDAs for other diseases under the NTD classification including onchocerciasis, trachoma, schistosomiasis and soil-transmitted helminthiases. Mass drug administration for lymphatic filariasis is based on annual application of a combination of ivermectin (150–200 µg/kg body weight) and albendazole (400 mg/person) to individuals in endemic areas aged ≥5 years. With the aim of improving the delivery of this intervention, the distribution of drugs is carried out following the Community-Directed Intervention (CDI) strategy, which aims to have communities themselves direct the intervention, following initial introduction to the intervention by local health workers and intervention partners. According to the CDI strategy, individuals are selected among, and preferably by, the community members to distribute drugs in their ‘communities’, meaning in the geographical vicinity of their homes, usually for a minor fee (Mutalemwa *et al.*, 2009; Kisinza *et al.*, 2008; CDI Study Group, 2010). Distributors are referred to as Community Drug Distributors (CDDs). In both rural and urban areas, CDDs visit other inhabitants' houses to distribute drugs to household members if they are at home. However, in some urban settings, drugs are also distributed in public places like institutions and market places. According to CDI requirements, staff from local health facilities are responsible for training the drug distributors, supplying drugs brought to them from the district offices, supervising drug distribution, collecting data and reporting to the districts. The implementation of MDA in Tanzania has been met with mixed reactions ranging from appreciation, to passive acceptance to outright refusal in various communities (Parker & Allen, 2013). Like in many other places, the drug uptake (compliance) rates have often been sub-optimal (Simonsen *et al.*, 2010, 2013; Kisoka *et al.*, 2014), but only a few studies have given attention to local perceptions of lymphatic filariasis and MDA (Allen & Parker, 2011; Parker & Allen, 2013).

This paper presents data from the qualitative component of a Tanzanian mixed-methods research project. That same project's quantitative component included a questionnaire-based cross-sectional household survey carried out in two rural and two urban sites in Tanzania, shortly after the 2011 MDA campaign for lymphatic filariasis (Kisoka *et al*., 2014). In this survey, 3279 adults were interviewed about MDA drug uptake among themselves and their children and asked their reasons for taking or not taking drugs. The study reported an overall drug uptake rate of 55.1% (range of 44.5–75.6% between sites) and that the main reasons for not taking the drugs were that people were either not at home at the time of distribution, or that they had not been offered the drugs.

The qualitative research data reported in this paper are based on interviews and focus group discussions with community members from the same four sites in Tanzania shortly after they had been targeted for MDA for lymphatic filariasis. The primary focus was to gain insight into targeted community members' perceptions and experiences of lymphatic filariasis, the drugs distributed and the phenomenon of MDA so as to indicate ways of improving the intervention and the interaction between populations and the intervention for future campaigns. This insight may also be relevant to MDA campaigns for other neglected tropical diseases.

## The research sites

**Fig. 1.**
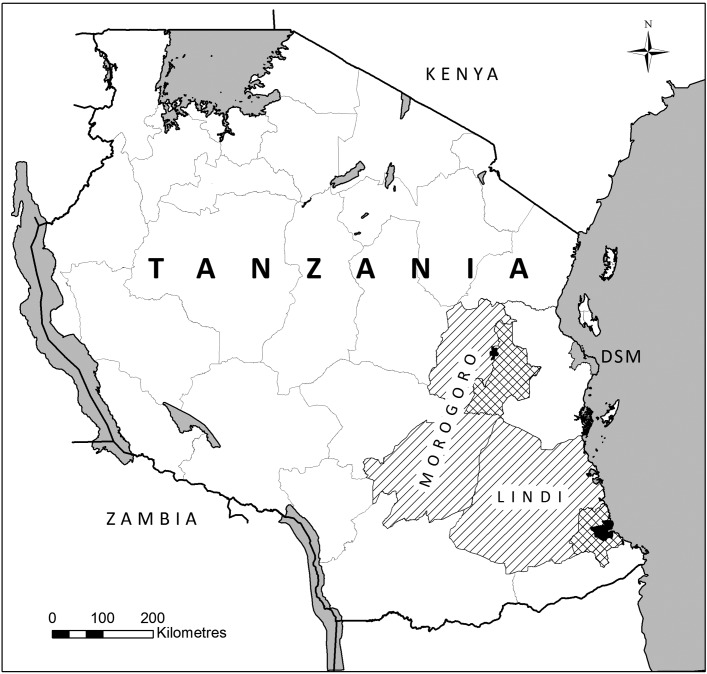
Map showing the location of the study sites in Lindi and Morogoro regions, Tanzania. Black: the two urban districts. Chequered: the two rural study districts. Hatched: the remaining part of the two study regions. DSM: Dar es Salaam.

The study was conducted in the Morogoro and Lindi regions of Tanzania (Fig. 1). According to the 2012 National Census, Lindi region had a population of 864,652, of which 78,841 lived in the regional capital of Lindi town, while Morogoro region had a population of 2,218,492, of which 315,866 lived in the regional capital of Morogoro town (NBS, 2013). From each region, one rural and one urban study site located at a considerable distance from each other were selected. In Lindi region, the rural site (in the following called Lindi Rural) was located in Lindi rural district about 75 km from the urban site in Lindi town (in the following called Lindi Urban), while in Morogoro region the rural site (in the following called Morogoro Rural) was located in Morogoro rural district about 150 km from the urban site in Morogoro town (in the following called Morogoro Urban).

The populations in the two rural study sites mainly comprise Makonde in Lindi Rural and Luguru and Kutu in Morogoro Rural, while in the two urban study sites the populations are rather mixed and also include people of Indian and Arab origin. However, while the population in Lindi Rural can be described as relatively homogeneous and stable, that of Morogoro Rural includes Maasai and Sukuma who have moved and settled in the areas in search of grazing land for their livestock. The settlement of these groups has been characterized by conflicts with the indigenous population over land use and occupancy. The great majority of the inhabitants of Lindi Region are Muslim, whereas in Morogoro region there is an approximate equal proportion of Muslims and Christians. There are no obvious religious tensions in the study sites.

In the rural areas, the majority of the residential premises are located along the main roads while the farms are located further inland. Houses are characteristically built of mud and poles and roofed with grass or thatch although there are a few built of bricks with corrugated iron roofs. People in the rural areas are involved in subsistence farming of food and cash crops. In Lindi Rural the main food crops are maize and cassava while cash crops include groundnuts and cashew nuts. There is also small-scale fishing. In Morogoro Rural the main food crops include maize, banana, rice, sorghum and beans while cash crops include onions and sugar cane. There is also livestock keeping of cattle, goats and chickens. In both Lindi and Morogoro Urban people are mainly employed in formal and informal sectors, including civil service, private sector employment and business. There is industrial sector production in Morogoro Urban while in Lindi Urban people are also engaged in small-scale fishing.

Administratively, the districts in Tanzania are divided into wards. Six of thirteen wards in Lindi Urban were selected for the study (four central and two peri-urban: Rahaleo, Matopeni, Nachingwea, Mwenge, Mtanda and Msinjahili) while three of nineteen wards in Morogoro Urban were selected for the study (one central and two less central: Kingo, Kichangani and Kingolwira). The four central wards selected from Lindi Urban were located close to the headquarters of the municipal council and regional headquarters. The regional referral hospital for Lindi and three dispensaries were easily accessible for residents of these wards. Each of the two peri-urban wards from Lindi Urban also had a dispensary. One of the wards selected from Morogoro urban was centrally located compared with the other two, although none of them could be classified as peri-urban. Residents in these wards were all within easy reach of health services. One ward was selected for the study in each of Lindi Rural (Nachunyu) and Morogoro Rural (Mngazi). There was a health centre in Nachunyu and a dispensary in Mngazi ward.

## Methods

The study was qualitative and comprised of interviews and focus group discussions (FGDs) held at the four study sites. These activities were carried out within one week after MDA for lymphatic filariasis had been completed at the study sites (i.e. in Lindi in May 2011 and in Morogoro in August 2011).

The interviewees comprised 21 CDDs, evenly distributed according to sex. The age range of CDD participants was 20–53 years, and most were in their 40s. Four male and five female CDDs were from Morogoro Rural, and two male and four female CDDs were from Morogoro Urban. In Lindi Rural only one male CDD was interviewed, while in Lindi Urban three male and two female CDDs were interviewed. Half of the interviewed CDDs reported that they participated in the MDA for the first time, while the others had participated one or more times previously. Eleven community leaders, all males, were interviewed. Of these, four were from Morogoro Rural, two from Morogoro Urban, three from Lindi Rural and two from Lindi Urban. Six religious leaders, all males, were also interviewed. Three were from Morogoro Rural, one from Morogoro Urban and two from Lindi Rural, while there was none from Lindi Urban. Health workers manning health facilities serving communities in study areas provided information that mutually contrasted, complemented and contextualized community perception on health problems and health services.

Eighteen FGDs with community members representing groups of adults and adolescents of both sexes were organized. There were five group sessions with young males (two in each district except Morogoro Rural where only one session was organized), five with young females (one in each district except Lindi Urban where two sessions were held) and four sessions for adult males and four sessions for adult females, one in each district. Each group comprised 8–12 discussants who were invited by community leaders one or two days prior to the discussions. The interviews and discussions took place in convenient places where there was no interference from passers-by. The interviewing and FGD process took cognizance of the fundamentals of collecting qualitative data, including promoting relaxed and trusting relationship with informants and FGD participants, encouraging participation, observing non-verbal cues, probing, noting silences, taking notes and opening and closing the interviews.

Interviews and FDGs were conducted in Swahili. Data collection tools were field-tested in a separate community before they were applied to the field. They solicited information from the research participants regarding the most pressing health problems faced by community members within their areas, health services available within their residence and their experience with health services. The inquiry into their perception of MDA for lymphatic filariasis sought information on the importance they attached to lymphatic filariasis and about their awareness and perception of lymphatic filariasis and the MDA process.

### Data analysis

All field notes including recorded interviews and conversations from FDGs were transcribed verbatim and translated by trained social scientists. It is recognized that transcription is not merely aimed at capturing the words of the participants, but also meanings and perceptions that lend contexts and explanations to responses and behaviours (Kvale, 1996). Transcribers were trained to avoid summarizing statements but instead to loyally represent slang, jargons, murmurs and sighs.

Transcribed data were carefully read and re-read by the research team in order to gain a full overview of the field data. Using thematic content analysis the research team performed multiple-level data coding (Graneheim & Lundman, 2004). This was done in an attempt to create codes that as closely as possible reflected the content of the data rather than researchers' pre-conceptions. Concepts used by the informants rather than the questions raised in the interview guide were employed as codes. The coding categories extracted from the transcripts and field notes were used to systematically analyse topics that were repeatedly mentioned in making up patterns of informants' opinions and experiences. Concurrently, attention was paid to contradicting views and experiences to reflect variations emerging in the data.

### Ethics

Research and ethical clearance for the study was provided by the Medical Research Coordinating Committee (MRCC) of the National Institute for Medical Research, Tanzania (reference number NIMR/HQ/R.8a/Vol. IX/1073). Study purpose and procedures were explained to study participants before they gave informed verbal consent to participate.

## Results

In this section the perceptions and concerns of the study population are presented under the following four headings; perceptions of major health problems among community members; concerns over the state of health care services; perception of lymphatic filariasis; and, finally, perceptions concerning MDA.

### Perceptions of major health problems among community members

With the purpose of contextualizing MDA for lymphatic filariasis within the general experience of health and health care among community members, FGDs included a focus on participants' perception of the most common serious health problems they experience in the community (Table 1). Among health workers, the question was asked in order to find out if there was common ground with community members with regard to health problems and experience with provision of health services.

**Table 1.** An overview of the major health problems mentioned by community members during focus group discussions in rural and urban study sites, Tanzania, listed according to priority with those considered more severe first

	Area of residence			
Informants	Lindi Rural	Morogoro Rural	Lindi Urban	Morogoro Urban
Boys	STDs^a^ Cholera Malaria	Malaria Cholera Skin diseases Lymphatic filariasis Eye diseases	Malaria Diabetes Stomach ulcers Cholera Lymphatic filariasis	Eye diseases Malaria Typhoid Worms Malnutrition
Girls	Malaria Diarrhoea Coughing Polio	Cholera Skin diseases	Malaria Lymphatic filariasis Stomach problems Diabetes Cholera Polio	Malaria Schistosomiasis Typhoid Lymphatic filariasis
Men	Malaria Cough Pneumonia Injuries Cholera	Eye problems Fever Skin diseases Malaria Worms Cough	Malaria, Cholera Stomach problems Lymphatic filariasis	Worms Malaria Typhoid Diarrhoea Cough
Women	Malaria Diarrhoea & vomiting Eye diseases Stomach ulcers Respiratory infections	Fever Malaria	Malaria Diarrhoea Diabetes Cancer Hypertension Cough	Malaria Diarrhoea Lymphatic filariasis

a Sexual transmitted diseases.

Community members were most concerned about diseases related directly to mortality as compared with those associated with morbidity. It is not surprising, therefore, that malaria was mentioned in all but one focus group discussion. A group member said:

Malaria is the major disease that disturbs us especially during the rainy season. Although we were given insecticide treated nets, malaria is still a big problem because the nets are for use in our beds but what about when we are outdoors especially during evenings how can we avoid the mosquitoes?

(Adult male, Lindi Urban)

A discussant in another group echoed this statement when she said:

Malaria is a big problem because our environment is characterized by mosquitoes.

(Adult female, Morogoro Urban)

Environmental health concerns, including water-borne diseases such as diarrhoea, typhoid and cholera, were mentioned in all groups from Lindi Region and a few from Morogoro region.

As far as health problems are concerned in our area, I am not very far from my fellows; another problem is lack of clean and safe water, therefore we are exposed to outbreaks.

(Adult male, Lindi Urban)

Eye problems and skin diseases were only mentioned by groups from Morogoro, while STDs were only mentioned by a group of young men in Lindi Rural.

Lymphatic filariasis was mentioned in five groups, four of which were in urban settings. In one of these groups discussants acknowledged it to be a big problem:

Lymphatic filariasis is a major problem here because people who have swollen legs and hydrocoele exist in this area.

(Adult women, Morogoro Urban)

In all groups where lymphatic filariasis was mentioned, hydrocoele was seen as a more prevalent complication than elephantiasis. Most interviewed community leaders were of the opinion that lymphatic filariasis was not a big problem within their areas, although they acknowledged hydrocoele had occurred:

Lymphatic filariasis is not a big problem in my area. There is nobody with elephantiasis. There was only one person who passed away several years ago. But those with hydrocoele exist. It is just a common thing.

(Muslim religious leader, Morogoro Rural).

Health workers confirmed the health concerns of community members regarding infectious diseases and shortages of essential medicine as threats to their health, and also mentioned poor environmental health and sanitation. One health worker said:

There is a big problem of water shortage and thus outbreaks of diarrhoea, so they would need an intervention on that.

(Lindi Urban)

Health workers also mentioned health system constraints that concerned community members:

The main complaint from people here is lack of laboratory services for diagnosing diseases. People do not want to be given malaria drugs if they are not properly diagnosed using laboratory equipment.

(Lindi Rural)

Health workers did not mention lymphatic filariasis among health problems they attend to in their daily activities, although through participating in the MDA interventions they acknowledged its presence.

### Concerns about the state of health care services

In both urban and rural settings the health service was perceived to be extremely poor and in every FGD participants lamented the poor state of local health services available to their communities. This situation is made worse when community members are forced to pay for poor quality services. In Morogoro Rural a young woman said:

If you go to the health facility, they ask you if you have health insurance. You say ‘yes I have’, but they will tell you, there are no medicines. They give you a prescription and tell you to go and buy medicines, while you have a health insurance. When is the government going to bring medicines to our health facility?

Another participant chipped in:

It is true as the previous participants have just said. We don't know how you will help us; we are facing a big problem that sometimes people are left to die.

An adult woman from a rural community remarked:

We are not satisfied because for women who attend that facility during delivery, it is a problem. Diagnostic equipment is not available, so we get treatment by guessing.

(Morogoro Rural)

Such despair was echoed in Lindi region where two FGDs with men emphasized their experience of poor political support for advocacy for better health care. One adult man in Lindi described it this way:

There is nothing we can do. Even when we raise our concerns with the higher authorities, the only thing we get from them are promises to visit us. Our village executive officer is only allowed to visit ward offices, he is not allowed to go any further. It is the councillor who has to take up the matter at the district but the councillor does not care about our problems, he only goes for his own things. How come the councillor does not visit his people for more than four months?

When asked about what therapy is available for lymphatic filariasis, community members mentioned traditional medicine as well as biomedical hospital-based surgery for those affected by hydrocoele. In one focus group in Morogoro, a young female participant mentioned that some people with elephantiasis visited traditional healers. In another group a discussant remarked:

There are a few cases of elephantiasis but many people have hydrocele. I am one of them. I have been getting treatment at Mtwara.

(Adult male, Lindi Rural)

In a group of women this was also raised when a participant remarked:

Yes, there are traditional medicines – people say they dig up the roots. If the swelling is at early stages it disappears.

### Perceptions of lymphatic filariasis

There is no single Swahili word for lymphatic filariasis infection. The condition is generally referred to by its common chronic disease complications of elephantiasis and hydrocoele. The official Swahili translations for the two complications are *matende* and *mabusha*. Hydrocoele is sometimes also referred to as *ngiri* *maji* or *mshipa*; the latter word may also refer to hernia (sometimes people tend to refer to hydrocoele as *mshipa wa kushuka* to distinguish it from hernia). In none of the FDGs was lymphatic filariasis mentioned as a major health problem despite the MDA programme. Informants acknowledged the presence of people who had either elephantiasis or hydrocoele within their localities when they were asked. Informants in all discussions and interviews shared the view that hydrocoele was a more important problem than elephantiasis because more people had the former complication. In some places people thought elephantiasis was non-existent in their communities:

There are no people with elephantiasis here, but a few with hydrocele.

(Adult woman, Lindi Rural)

One community leader shared this view:

Lymphatic filariasis is not a major problem in my area. There is nobody with elephantiasis. But those with hydrocoele exist.

(Religious leader, Morogoro Rural)

A community drug distributor voiced the same opinion when he said:

The problem of elephantiasis is not there, because no one is suffering from it but there are a few who are suffering from hydrocoele, because our environment is attractive to hydrocele.

(Morogoro Rural)

Elephantiasis and hydrocele were assigned various causes by informants. The MDA campaigns aim to inform community members that the tablets distributed by the programme are meant to treat the infection that causes both these manifestations. The diversity in the perceptions of the cause of lymphatic filariasis manifestations by community members is a reflection of the dysfunction in the communication strategy. This point was provided by one of the distributors, who said:

From what I learned in school it is transmitted by mosquitoes, but we were not told so in this MDA.

(Morogoro Urban)

In many instances where mosquitoes were referred to, some community members thought they only caused elephantiasis. Hydrocoele was seen to be due to a variety of other causes:

There are mosquitoes of a particular species which bite people and cause elephantiasis. We don't know what causes hydrocoele. Perhaps men themselves can be good experts in that because this happens in their environment.

(Adult female, Lindi Urban)

Also, among drug distributors, the perceptions with regard to causes of lymphatic filariasis chronic manifestations (hydrocoele and elephantiasis) varied. This was despite the fact that one aspect in their training related to cause and transmission of lymphatic filariasis, which they were in turn supposed to communicate to community members during sensitization campaigns:

As far as I know hydrocoele is that disease which a man can get as a result of filling up with liquid. We call it *ngiri maji.* Elephantiasis is a disease which a person gets from worms that are found in the water. The person gets the disease by entering into the water and being bitten by insects living in the water with worms.

(CDD, Lindi urban)

One CDD, however, had another idea about the aetiology of lymphatic filariasis:

I understand about this problem because I have been involved in drug distribution for about 3 years. I attended the seminar and have learned that lymphatic filariasis is transmitted by a mosquito called *Culex* and worms are the results of eating fruits that are not washed properly and poor use of toilets.

(Morogoro Urban)

The difference in understanding of the causes of lymphatic filariasis manifestations among drug distributors reflects the lack of depth in the training provided to this important group of stakeholders in the MDAs.

Community leaders, regardless of their areas of residence, also demonstrated that understandings of lymphatic filariasis varied, especially with respect to its cause and mode of transmission. This is also a reflection of weaknesses in communication strategies among programme implementers. Community leaders are an important link in the communication pathway between implementers at higher levels and recipient community members. Any distortion at this level is bound to result in misunderstandings and lack of appropriate knowledge.

### Perceptions of MDA

Overall, community leaders and drug distributors perceived MDA activity positively. They generally expressed trust in the fact that MDA was beneficial to individuals and communities. The benefits mentioned were related to lymphatic filariasis disease prevention, cure or rehabilitation of several conditions. In one community, the village leader gave an example to emphasize this point:

My child was suffering from skin itching but after using these drugs, the problem ended.

(Community leader, Morogoro Rural)

One of the community leaders put it succinctly when he said:

Yes there are benefits; if a person is infected the drugs kill the infection and at the same time build immunity. If taken for five years the disease is eradicated completely. The mosquitoes that transmit the disease will no longer be able to do so; eventually there will be no more infections.

(Community leader, Lindi Rural)

Another community leader thought that the MDA was good because drugs were being given to people free of charge:

Because they are provided freely, instead of using money to buy, it is good.

(Morogoro Rural)

Drug distributors gave almost the same account as community leaders with respect to how they perceived the mass distribution of drugs. Their reservations, however, were on the challenges they faced with the task of distributing drugs to community members. The challenges mentioned include diverse reactions they encountered from the community members, some of whom believed they had no problems for which treatment was being provided, little or no trust from community members who thought CDDs were not up to standard when handling medicine, as well as the difficulties of reaching them given the terrain, distance, short time allocated and timing with the distribution. The perceptions of CDDs of the MDA were summarized in these terms:

MDA has contributed to reducing problems associated with lymphatic filariasis.

(Morogoro Urban)

There were also appreciative reactions to MDA among community members, for instance participants said:

I used to have filarial attacks but now I don't have them.

(Adult male, Lindi Rural)

and:

The drugs are both preventive and curative. For people not yet infected when they take these drugs they don't get the disease, for those with infections but no symptoms the parasites are killed. For those with early symptoms of the disease the symptoms disappear.

(Young female, Lindi Urban)

But there was also a lot of scepticism in relation to MDAs, and the aims of drug distribution. A FGD participant explained:

Individuals benefit by taking drugs because they are for prevention, except that the circumstances under which the drugs are distributed are doubtful. There was a time when a tailor died and people said it was because of these drugs.

(Young female, Lindi Urban)

In Morogoro Rural, community members thought the drugs were provided to control onchocerciasis because ivermectin had been used for several years to control onchocerciasis before.

We heard about those drugs for onchocerciasis and albendazole. They were brought by selected distributors.

(Adult female, Morogoro Rural)

In some instances community members were reportedly protesting against treatment for the disease, which was not their priority. One CDD recounted how he was rebuked when he visited a house:

We don't want your drugs. Instead of bringing us important things you come with drugs.

(Lindi Urban)

And yet still, some members of the community expressed their scepticism about the programme. This was partly due to side-effects experienced or perceived by some community members. For instance, one discussant had this to say:

Those who refused drugs had several reasons. Previously they took the drugs and they became seriously ill so they associated the illness with the drugs.

(Young female, Lindi Urban)

Fears that drugs were really aimed at harming people, particular male sexual potency, were not uncommon, as was observed by one CDD:

Problems were that some people were refusing to accept the drugs due to the belief that drugs cause male impotence and when the father refuses, all people in that household also refuse.

(Morogoro Urban)

These misconceptions were also observed with respect to lymphatic filariasis being a disease for rural areas rather than the population in urban areas.

Many people have the problems but when we tell them about drugs they refuse saying this disease is for rural areas and not urban areas.

(CDD, Morogoro Urban)

An older woman, representing community feelings of conspiracy, said:

We don't trust free drugs; they have been brought to finish us off. People believe that these drugs have a hidden agenda that is the main reason; other reasons are just excuses. Free drugs are brought to kill us. People are afraid to use even the free bed nets provided. They don't use them to protect themselves or their children against mosquitoes but rather they use them to store their harvest.

(Lindi Rural)

Experiences with, or rumours about adverse effects of drugs were cited by CDDs and community members as being among the reasons for low drug uptake. A range of effects were reported by those who had been affected and/or whose relatives, or people they knew, had been affected by taking drugs. The perceived effects included fever, dizziness, vomiting, nausea, severe itching and swelling of different parts of the bodies. The lack of explanation for the adverse effects most likely fuelled the existing rumour about drugs and the aim of distribution.

Those who refused drugs had several reasons. They said drugs kill. Previously they took the drugs and they got seriously ill so they associated their experiences with the drugs. Some of them claimed the drugs were for experimentation. Others say that we are given the drugs so that we don't reproduce.

(Young female, Lindi Urban)

In some communities, the distribution process was said to have contributed to the MDA being perceived negatively. The involvement of community members in the distribution of drugs was not a new phenomenon in the study districts. However, it was observed that some community members were not pleased with this practice as they felt they could not trust people whom they knew to have never received even basic training in handling drugs. As one discussant put it:

Nurses and health workers should be used to do the distribution, not these community members who have caused many to refuse taking the drugs.

(Young female, Lindi Urban)

Some of community members were of the opinion that the practice of measuring heights of individuals instead of their weight as the basis for determining dosage for ivermectin was one of the reasons for their doubts and refusal to take drugs. One female participant voiced this concern when she said:

This practice should be changed. It is better to measure weight for dosage because using height a child may take more tablets than the father and therefore cause misunderstanding.

(Young female, Lindi urban)

## Discussion

The present study formed part of a larger project that aimed to identify barriers and strategies for improving drug coverage in rural and urban areas under the MDA programme for lymphatic filariasis control in Tanzania. A quantitative household survey carried out in parallel with the present study established that the overall drug uptake rate at the study sites in 2011 was only 55.1% (Kisoka *et al.*, 2014). According to WHO, coverage is required to be a minimum of 65% of the population to break transmission over a period of 5–6 years (WHO, 2011). However, mathematical modelling has shown that a higher coverage and/or longer treatment periods may be required in areas with very high pre-control lymphatic filariasis prevalence rates (Michael *et al.*, 2004). Efforts to increase coverage need to examine pertinent dimensions of the relationship between the intervention and the targeted communities. Therefore in this study, the focus was on the populations' perceptions of, and experiences with, MDA in the context of the health concerns and health care challenges that communities face.

A recent literature review by Krentel *et al.* (2013), focusing on factors that affect individual compliance with MDA for lymphatic filariasis, mentioned five key ingredients in promoting success: attention to trust issues, adaptation to local conditions, minimization of adverse effects of the drugs, promotion of the broader benefits of the MDA programme and addressing the challenge of systematic non-compliance. Parker & Allen (2013) aimed to turn attention towards the international players' responsibility for changing this scenario rather than continuing to focus on the ‘hearts and minds’ of the communities targeted for treatment. Among such international players are pharmaceutical companies, which play a role, for better or worse, in MDA interventions (Samsky, 2011, 2012).

The CDI strategy has, in the last decade, increasingly been applied with a view to addressing challenges to access to, and coverage of, MDA-based interventions through promoting community mobilization, participation and ownership. It was developed and applied in 1995 to strengthen community involvement and ownership and ensure that rural and remote villages received annual doses of ivermectin to control onchocerciasis. Community-directed intervention has since been applied to increase access to malaria treatment, Vitamin A and deworming medicine (CDI Study Group, 2010). The CDI strategy has much potential but MDA through the CDI strategy faces a number of challenges. A central component of MDA implemented through the CDI strategy is the emphasis on advocacy and mobilization among local stakeholders and, furthermore, the emphasis on information and communication about the campaign and its aims and means (CDI Study Group, 2010). This process should ideally allow for a genuine dialogue, including in-depth information sharing and discussions on various aspects of the intervention (Amazigo *et al.*, 2012). The programme staff and implementers' approach to information, education and communication about lymphatic filariasis and MDA should take centre stage in the implementation process, preferable supported by primary and secondary school curriculum and adult education programmes. This is both a costly and hugely complex task, but necessary for a more successful intervention.

A good level of understanding of the disease's aetiology, development of the infection and its manifestations is important for peoples' acceptance of MDA, not just to dispel misconceptions and promote shared understandings of lymphatic filariasis and the distributed drugs. It is just as much a means of promoting ownership and dignified informed participation in the intervention. The population should feel that their views and concerns matter. Through communication, debate and education, positive citizenship and a sense of ownership and belonging is promoted. Unfortunately, the FGDs showed that people did not feel they were adequately informed during the campaign. In addition, the study indicated that there was considerable variation in how community members both within and across study districts perceived the MDA and distributed drugs; some community members appreciated the distribution of drugs, and requested the drugs from local health facilities if they missed the distribution. Others wished to avoid the drugs due to lack of confidence in the rationale for drug distribution or fears of adverse effects of the drugs. The relationship between the vector, the parasite and eventual disease manifestations is complicated and not easy to explain and understand and the study shows that there was considerable uncertainty and misunderstandings about this among community members. In this respect, the programme could consider identifying a proper Swahili term for lymphatic filariasis which should be descriptive enough to promote a better understanding of the fact that the serious disease complications of elephantiasis and hydrocele are both consequences of the lymphatic filariasis infection. Moreover, regular training targeting CDDs should be provided to enable them to inform and discuss with community members and address their request for adequate information, and NTD staff at national, regional and district levels should be refreshed on the principles and values of CDI and primary health care.

The review by Krentel *et al.* (2013) listed studies that reported good compliance with MDA of people who perceived themselves to be at risk of lymphatic filariasis infection, while another study observed that lack of tangible results of taking drugs raises suspicion about the actual aim of the programme (Parker & Allen, 2012). Most community members were familiar with the conditions of elephantiasis and hydrocoele because they were familiar with people who suffered from these conditions. In other communities, lymphatic filariasis was mentioned among health problems being faced by people, apparently because the research team had informed them of the purpose of the study. However, most community members were unfamiliar with the cause and mode of transmission of lymphatic filariasis; and the fact that elephantiasis and hydrocoele are manifestations of the same infection. This led community members to suggest that drugs should only be given to those with hydrocoele and elephantiasis or, alternatively, people should be screened to determine if they had infections so as to avoid dispensing drugs to people without evidence of infection. As one CDD explained:

We did not encounter many problems except that some people refused to take the drugs, saying that they would not take it, because they were not sick.

Manderson wrote that neglected tropical diseases exist for social and economic reasons and that persistent poverty and inequality contribute to their continued existence (Manderson *et al*., 2009). At the same time, impoverished population groups may not necessarily prioritize treatment for lymphatic filariasis as they are already facing a number of life-threatening conditions such as malaria and respiratory diseases, for which the required health care and interventions are inadequate at best or even non-existent (Bhullar & Maikere, 2010; Parker & Allen, 2012; Samuelsen *et al.*, 2013). Research participants expressed disappointment in the political leaders and their inability to stand up for their communities and promote positive change, leaving people feeling deserted and socially and politically marginalized. The failure to address such challenges as poor health care, the severe lack of essential medicines and social and political marginalization, which impact on the daily lives of poor communities, angered some focus group participants and, as noted above, led one community member to exclaim to a distributor:

We don't want your drugs. Instead of bringing us important things you come with drugs.

In spite of this dire opposition, there is huge and promising potential in the positive spirit with which some community members and drug distributors took part in MDA and received the drugs. One community leader made this point clearly when he said,

This is a gradual process which needs time to reach its target.

(Morogoro Urban)

The study documented positive viewpoints and a feeling of empowerment among some community members towards both the short-term effects of the drugs and also occasional positive and optimistic sentiments and understandings of the long-term potential of MDA to eliminate lymphatic filariasis.

It should also be noted that although many community members after drug distribution appeared to be discontented with the programme, it is not known to what extent this dissatisfaction resulted in an individual decision to *not* accept the treatment. A quantitative household survey in the same communities (Kisoka *et al.*, 2014) found that the overall drug uptake rate in 2011 was only 55.1%, but according to this study component, the main reason for this was not that people refused the drugs, but that they were never offered it during the campaign for various reasons. The study therefore concluded that improved drug uptake relied *more* on programme-related factors that are modifiable, than on perceptions and practices of the target population. This present study has identified other programme-related challenges that can and should be addressed by programme stakeholders. More importantly, as other social scientist have pointed out (Parker *et al*., 2008; Manderson, 2012), international, national and local institutions and power holders must pay attention to the needs and priorities of ‘neglected populations’ if MDA is to live up to its promises over time.

### Conclusions

While global and national health policy push for MDA for control of lymphatic filariasis as necessary to interrupt lymphatic filariasis transmission, community members and representatives who were given a voice in this study said that they had other, more pressing, concerns and they felt excluded from information and communication about the campaign. The programme, to a great extent, was unable to fully engage the recipient communities in the important stages of the process. While the NTD programme allegedly followed the community-directed approach to implementing MDAs, central elements of this policy seemed to be missing from the point of view of community members. To address the observed shortcomings, it is important that national-, regional- and district-level authorities take the community-directed approach seriously and involve communities from the early stages of MDA planning and implementation. Community mobilization and ownership form the backbone of community-directed distribution and these cannot develop if information and dialogue is not prioritized as an essential part of the process. The concerns and challenges of the neglected populations need to be taken into consideration to sustain and improve acceptability and support for the MDA-based control activities.
